# Functional connectivity of the hippocampus and its subfields in resting‐state networks

**DOI:** 10.1111/ejn.15213

**Published:** 2021-05-05

**Authors:** Laura Ezama, Juan A. Hernández‐Cabrera, Sara Seoane, Ernesto Pereda, Niels Janssen

**Affiliations:** ^1^ Facultad de Psicología Universidad de la Laguna La Laguna Spain; ^2^ Instituto de Tecnologías Biomédicas Universidad de La Laguna La Laguna Spain; ^3^ Instituto Universitario de Neurociencia Universidad de la Laguna La Laguna Spain; ^4^ Basque Center on Cognition Brain and Language San Sebastián Spain; ^5^ Facultad de Ingeniería Industrial Universidad de La Laguna La Laguna Spain

**Keywords:** CA1, Hippocampal subfields, ICA, Resting‐State fMRI, subiculum

## Abstract

Many neuroimaging studies have shown that the hippocampus participates in a resting‐state network called the default mode network. However, how the hippocampus connects to the default mode network, whether the hippocampus connects to other resting‐state networks and how the different hippocampal subfields take part in resting‐state networks remains poorly understood. Here, we examined these issues using the high spatial‐resolution 7T resting‐state fMRI dataset from the Human Connectome Project. We used data‐driven techniques that relied on spatially‐restricted Independent Component Analysis, Dual Regression and linear mixed‐effect group‐analyses based on participant‐specific brain morphology. The results revealed two main activity hotspots inside the hippocampus. The first hotspot was located in an anterior location and was correlated with the somatomotor network. This network was subserved by co‐activity in the CA1, CA3, CA4 and Dentate Gyrus fields. In addition, there was an activity hotspot that extended from middle to posterior locations along the hippocampal long‐axis and correlated with the default mode network. This network reflected activity in the Subiculum, CA4 and Dentate Gyrus fields. These results show how different sections of the hippocampus participate in two known resting‐state networks and how these two resting‐state networks depend on different configurations of hippocampal subfield co‐activity.

**Abbreviations**
ANOVA: analysis of variance
AP: anterior‐posterior
aparc: atlas for cortical parcellation
aseg: automatic subcortical segmentation
CFS: cerebrospinal fluid
CMRR: Center for Magnetic Resonance Research
DG: dentate gyrus
emmeans: estimated marginal means
EPI: echo‐planar imaging
FC: functional connectivity
FIX: FMRIB's ICA‐based X‐noiseifier
FOV: field of view
FSL: FMRIB Software Library
ggpubr: Based Publication Ready Plots
ggseg: plotting tool for brain atlases
HCP: Human Connectome Project
ICA: independent component analysis
ICs: independent components
IRB: institutional review board
lmerTest: tests in linear mixed effects models
MCFlirt: intra‐modal motion correction
ML: molecular layer
MNI: Montreal Neurological Institute
MR: magnetic resonance
MRI: magnetic resonance imaging
PA: posterior‐anterior
QC: quality control
rsfMRI: resting‐state functional magnetic resonance imaging
srICA: spatially‐restricted independent component analysis
SUB: subiculum
TE: echo time
TI: inversion time
TR: repetition time
WM: white matter

## 1 INTRODUCTION

One of the major neuroimaging discoveries of the past few decades is the observation that when a person is at rest, there are several sets of brain regions that become activated at different points in time (Smith et al., 2013; Fox et al., 2005; Van Den Heuvel & Pol, 2010). How these sets of regions connect to form whole‐brain networks is one of the central questions of modern neuroscience research. Here, we focused on a brain structure called the hippocampus, a key region in many neurological and psychiatric diseases (Andersen et al., 2006; Duvernoy, 2005). While a large number of anatomical and functional connectivity studies have suggested that the hippocampus plays a role in a resting‐state network called the default mode network (Aggleton, 2012; Blessing et al., 2016; Kahn et al., 2008; Libby et al., 2012; Qin et al., 2016; Ranganath & D'Esposito, 2001; Vincent et al., 2006), many aspects regarding the connectivity of the hippocampus to known resting‐state networks remain unclear. For example, what section of the hippocampus connects with the default mode network? Given the extant evidence on the long‐axis organization of hippocampal function (Fanselow & Dong, 2010; Moser & Moser, 1998; Poppenk et al., 2013; Ranganath & Ritchey, 2012; Strange et al., 2014), it is likely that only a specific section of the hippocampus fulfills this role. In addition, besides the regions of the default mode network, the hippocampus is typically associated with a set of anterior brain regions. However, it is unclear whether these regions form part of a known resting‐state network. Finally, the hippocampus has a complex internal structure that is composed out of various subfields with different cell morphology and projection targets (Aggleton, 2012; Insausti & Munoz, 2001; Rosene & Van Hoesen, 1977). Given proposed functional differences between the subfields (Marr, 1971; McClelland et al., 1995; Yassa et al., 2011), how do the different resting‐state networks depend on the different subfields? Here, we approached these issues using functional connectivity (FC) derived from a high spatial‐resolution 7T dataset from the Human Connectome Project (HCP). Using data‐driven techniques, we first determined the precise location of hippocampal activity hotspots along the hippocampal long‐axis, examined the FC between these hotspots and known resting‐state networks and then studied the configuration of hippocampal subfield activity underlying the observed resting‐state networks.

It is now well established that there is connectivity between the hippocampus and regions of the default mode network (Fox et al., 2005; Fransson, 2005; Greicius et al., 2004; see Buckner & DiNicola, 2019, for a recent review). For example, FC studies have shown that seeds placed in major default mode network regions like the precuneus and posterior cingulate cortex produce connectivity with the hippocampus (Fox et al., 2005; Fransson, 2005). However, the precise section of the hippocampus that connects with these regions remains poorly understood. Anatomical studies on hippocampal connectivity in rodents have demonstrated that there are differences in connectivity between anterior (ventral) and posterior (dorsal) sections of the hippocampus (Fanselow & Dong, 2010; Poppenk et al., 2013; Strange et al., 2014). Specifically, these studies have found that anterior (ventral) hippocampus has direct connectivity with the amygdala and that this section of the hippocampus connects with regions of the medial and lateral temporal lobe through polysynaptic pathways. In addition, posterior (dorsal) hippocampus connects with posterior midline regions like retrosplenial cortex, thalamus and mammillary bodies through the fornix pathways (Jones & Witter, 2007; Rosene & Van Hoesen, 1977). However, in humans, FC studies of hippocampal connectivity with default mode network regions have not found consistent results. Specifically, although a number of studies have found connectivity between seeds in posterior sections of the hippocampus and the regions of the default mode network (Adnan et al., 2016; Barnett et al., 2019; Kahn et al., 2008; Qin et al., 2016; Voets et al., 2014), other studies have concluded that seeds in anterior sections of the hippocampus connect with the default mode network (Blessing et al., 2016; Chase et al., 2015; Robinson et al., 2015; Vincent et al., 2006). Thus, while it is clear from these studies that the hippocampus connects with regions of the default mode network, the precise section of the human hippocampus that is responsible for this connectivity remains unclear.

In addition, a common proposal is that besides the default mode network, the hippocampus has connectivity with a second brain network (Aggleton, 2012; Kahn et al., 2008; Kahn & Shohamy, 2013; Ranganath & Ritchey, 2012). This so‐called anterior network has been found to be composed out of regions such as the amygdala, nucleus accumbens, orbitofrontal cortex and temporal pole (Aggleton, 2012; Kahn et al., 2008; Kahn & Shohamy, 2013; Ranganath & Ritchey, 2012). In FC studies, it has been observed that seeds in anterior and middle sections of the hippocampus connected with areas in this anterior network (Blessing et al., 2016; Kahn et al., 2008; Kahn & Shohamy, 2013; Qin et al., 2016). However, although these studies revealed that the hippocampus is likely connected to a second anterior network, it is not clear whether this network can be associated with a previously identified reference resting‐state network (e.g., Yeo et al., 2011). Linking this second anterior network to one of the known reference resting‐state networks would enable a more broad interpretation of this network and may produce further insight into hippocampal function during the resting state.

**FIGURE 1:**
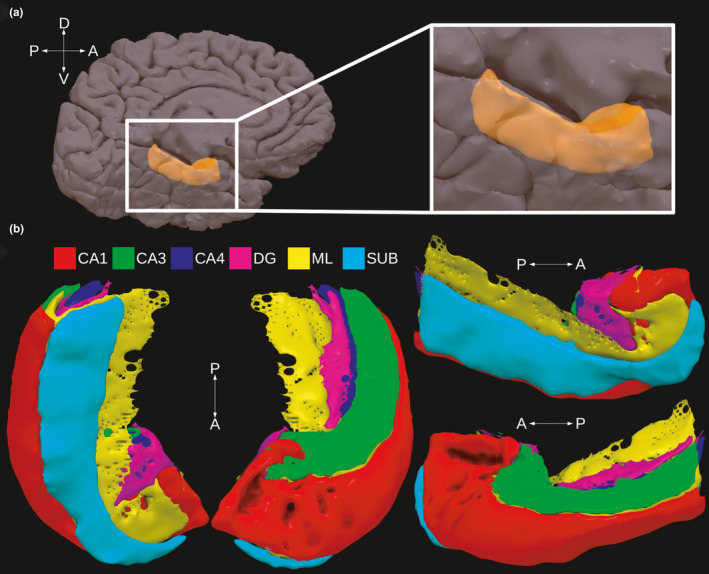
Presentation of hippocampal anatomy in a given participant from the experiment. Panel A shows the location of the hippocampus (in orange) on the medial surface of the brain. Panel B shows the various hippocampal subfields that were detected using automatic segmentation procedures (see text for details)

Finally, it is unclear how the different resting‐state networks putatively associated with the hippocampus depend on its internal structure. The hippocampal formation t the Cornu Ammonis (CA1, CA2, CA3 and CA4), the Dentate Gyrus (DG) and the Subiculum (SUB) subfields. These subfields are relatively small (in humans ∼1 mm in cross section; Insausti & Amaral, 2003), and furthermore, they show an intricate pattern of folding that is preserved along the long‐axis of the hippocampus (see Figure 1). The differences in cell morphology and connectivity of these subfields have motivated proposals regarding their functional differences (Marr, 1971; McClelland et al., 1995; Yassa et al., 2011). In line with such proposals, previous studies in monkeys and rodents have underscored the differences in anatomical connectivity between the CA1 field on the one hand, and the SUB on the other (Aggleton, 2012). Specifically, these studies have shown that anterior areas such as the amygdala, orbitofrontal cortex and nucleus accumbens, as well as medial and lateral temporal areas depend more on connectivity with the CA1 subfield, whereas posterior midline areas such as the retrosplenial cortex, anterior thalamic nuclei and mammillary bodies are connected to the SUB (Aggleton, 2012; Rosene & Van Hoesen, 1977). In humans, a handful of studies have examined FC between the hippocampal subfields and other brain regions (Dalton et al., 2019; de Flores et al., 2017; Shah et al., 2018; de Wael et al., 2018). However, the small‐sized subfields pose technical challenges in MRI acquisition that have placed limits on the ability to distinguish fMRI activity between the individual subfields or on the ability to acquire images with whole‐brain coverage (Carr et al., 2010). For example, some studies have looked at connectivity between the hippocampal subfields and regions within the medial temporal lobe (Dalton et al., 2019; Shah et al., 2018), whereas others used seeds in the hippocampal subfields that did not distinguish anterior from posterior hippocampus (de Flores et al., 2017). Thus, these studies do not allow for a clear conclusion regarding the connectivity between subfields in anterior and posterior sections of the hippocampus and the rest of the brain, and consequently, the way the hippocampal subfields participate in known resting‐state networks remains unclear.

In short, whereas it is clear that the hippocampus connects to the default mode network, it remains unclear which section of the hippocampus connects to this network, whether the hippocampus connects to other resting‐state networks, and how hippocampal subfields participate in the different resting‐state networks potentially connected with the hippocampus. In the current study, we addressed these issues with an analysis approach that relied on four main steps. First, we relied on a data‐driven approach called spatially‐restricted group ICA to pinpoint the exact location of hippocampal activity hotspots during the resting state (Blessing et al., 2016; Formisano et al., 2004). We used a novel method to determine the optimal number of dimensions for the ICA. This data‐driven approach offers advantages over a traditional seed‐based approach in which FC is calculated from seeds at brain locations that may or may not show activity in the resting state (see Zuo et al., 2010, for discussion). Second, we computed whole‐brain FC maps from these hippocampal activity hotspots using Dual Regression (Nickerson et al., 2017). These whole‐brain FC maps therefore reflected the FC between the hippocampal hotspots identified in the previous step and the rest of the brain. Next, we correlated these whole‐brain FC maps with the seven reference resting‐state networks of Yeo et al., (2011) to examine the relationship between the activity hotspots detected inside the hippocampus and the resting‐state networks in which they participate. Third, we computed group‐level contrasts between the different FC maps using linear mixed‐effect regression analyses that took into account the unique brain morphology of individual participants. Finally, we examined how the different hippocampal subfields contributed to the different resting‐state networks detected in the previous step. For this, we performed additional regression analyses of the individual participant's FC maps. Data analyses relied on the 7T dataset that is publicly available from the HCP (Van Essen et al., 2013). The 7T HCP dataset had 1.6 mm isotropic resolution and provided coverage of the whole brain. This dataset therefore leverages the high spatial resolution necessary to compare signals from the hippocampal subfields with signals from the rest of the brain (Carr et al., 2010). All analyses were performed at participant‐native resolutions that minimized group blurring.

## 2 Methods

### 2.1 Participants

The HCP dataset consists of a large sample of participants with 3T acquisitions (*N* > 1,100) and a smaller sample with additional 7T acquisitions. The full HCP 7T dataset consisted of 184 participants. Participants that did not meet specific HCP defined QC issues were removed from the study. Specifically, we removed those participants with anatomical anomalies (QC code A), segmentation problems in the structural pipeline (QC code B), head coil instabilities (QC code C) and prominent artefacts in the resting‐state scans (QC code D). This resulted in the final set of 172 participants that were used for our analyses. Within this set, 104 participants were female, and the most common age range was between 26 and 30 years (81 participants). The study was approved by the local IRB Committee (Comité de Ética de la Investigación y de Bienestar Animal) of the Universidad de La Laguna (CEIBA2017‐270).

### 2.2 MRI acquisition parameters

As per the HCP reference manual, the functional data were collected on a 7T Siemens Magnetron scanner located at the Center for Magnetic Resonance Research (CMRR) at the University of Minnesota in Minneapolis, MN. The scanner uses the Nova 32‐channel Siemens receive head coil with an incorporated head‐only transmit coil that surrounds the receive head coil from Nova Medical. Volumes were acquired using Gradient‐Echo EPI. Each volume contained 85 slices that were acquired with a multiband factor of 5. Slice thickness was 1.6 mm with no gap, the FOV was 208 × 208 mm, and matrix size was 130 x 130, resulting in 1.6‐mm isotropic voxels. The TR was 1,000 ms, echo time (TE) 22.2 ms, and the flip angle 45°. In each run, 900 volumes were collected and lasted around 16 min. Runs alternated between phase encoding in the posterior‐anterior (PA) and anterior‐posterior (AP) direction. Head motion, cardiac and respiratory signals associated with each scan were not collected. Although four resting‐state runs were collected for each participant, the current analyses used data from only the first two sessions (i.e. 32 min of resting‐state per participant). This was done to limit the time and storage requirements of the analyses, as well as because previous studies have demonstrated high test–retest reliability with rsfMRI scan durations of more than around 30 min per participant (Birn et al., 2013; Noble et al., 2017). During data acquisition, participants had to keep their eyes open by looking at a cross‐hair on a dark background.

In addition, structural T1w and T2w images were available for each participant. Again as per the HCP reference manual, these images were acquired on a customized 3T Siemens Connectome Skyra scanner. The T1w images were acquired using a 3D‐MPRAGE protocol TI/TR/TE: 1000/2400/2.14 ms, flip angle = 80°, resulting in 0.7 mm isotropic voxels. The T2w images were acquired using a 3D T2‐SPACE protocol TR/TE: 3200/565 ms, flip angle = variable, and also resulting in 0.7 mm isotropic voxels.

### 2.3 Preprocessing

We downloaded the Resting State fMRI 1.6mm/32k FIX‐Denoised (Compact) and Resting State fMRI FIX‐Denoised (Extended) datasets for each participant. The datasets that we downloaded therefore consisted of already preprocessed functional data according to HCP minimal preprocessing pipelines (Glasser et al., 2013). Without going into details, a brief summary of these preprocessing steps is as follows. First, transformations that reduced head motion were estimated using FSL MCFlirt, fieldmap and gradient distortion corrections were applied, and transformations from fMRI space to MNI space were estimated using non‐linear transformations. In order to minimize smoothing of the data due to repeated transformations, all these transformations were postponed, combined and applied in a single step using sinc interpolation. Next, the data in MNI space were temporally filtered using a 2000 s high‐pass filter and automatically denoised using the FIX program (Griffanti et al., 2014; Salimi‐Khorshidi et al., 2014). Note that there is a long‐standing discussion about the best method to deal with head motion in resting‐state fMRI data (Van Dijk et al., 2012; Friston et al., 1996), and that generally speaking, ICA based methods like FSL FIX perform among the best on this issue (see Griffanti et al., 2014, for comparisons). We therefore think that head‐motion issues were minimized in the HCP dataset. The final files were demeaned and had 1.6 mm isotropic resolution in MNI space. We then obtained participant‐specific masks for CSF and WM from the wmparc atlas supplied by FreeSurfer and regressed out the signal from CSF and WM from the fMRI files.

For the structural data, we downloaded the 3T Structural Preprocessed and 3T Structural Preprocessed Extended packages. These packages contained the T1w and T2w images for each participant as well as the full FreeSurfer output and transformation matrices that were relevant for our downstream analyses (see below). For specific information on the preprocessing of these structural images, we refer to Glasser et al., (2013).

### 2.4 Analyses

The analyses were applied to the cleaned HCP dataset in four main steps: First, the segmentation of the hippocampal subfields; second, the detection of activation clusters inside hippocampus and their relationship with resting‐state networks; third, group analysis of the obtained whole‐brain connectivity maps; and fourth, an analysis of the relative contributions of the hippocampal subfields that underlie the obtained resting‐state networks.

#### 2.4.1 Segmentation of hippocampal subfields

The full output from Freesurfer v5.3 (Dale et al., 1999) was available for each participant in the dataset. Automatic segmentation of the hippocampal subfields was performed on this output using the Hippocampal Subfields and Nuclei of the Amygdala script (v21) with the 0.7 mm T2w image as the input (Iglesias et al., 2015). We focused here on five subfields, namely, CA1, CA3, CA4, Dentate Gyrus (DG) and the Subiculum (SUB). In addition to these five standard subfields, we also included the substructure Molecular Layer (ML; see Figure 1 for anatomical details of the structures involved). The ML is a cell‐free layer that is easily identified in histology and MR images. We included the ML in the regression models (described below) because we hypothesized it would produce more accurate statistics. The ML occupies a key position with respect to the other subfields because it makes close contact with almost all other subfields (see Figure 1; see also Iglesias et al., 2015), and therefore including this subfield in the analyses produces (partial) estimates for the major subfields that are more accurately localized. In addition, note also that for reasons of lack of sufficient contrast in MRI, it was difficult to separate CA2 and CA3 regions, and therefore, the CA3 region also included the CA2 region (Iglesias et al., 2015). Finally, all automatic segmentations were visually checked for QC issues by identifying the overlap between the dark band in the T2w image and the detected ML by the segmentation algorithm. No issues were detected in this way.

#### 2.4.2 Detection of activation clusters inside hippocampus and relationship with resting‐state networks

In the next step, we attempted to find those areas of the hippocampus that were activated in the context of existing resting‐state networks. To this end, we first transformed each participant's aparc + aseg atlas from FreeSurfer space to HCP's MNINonLinear space with nearest neighbour interpolation. We then multiplied the participant's cleaned and denoised 4D fMRI data in MNI space by the bilateral and binarized version of the participant‐specific hippocampal mask extracted from the aparc + aseg atlas. This operation therefore produced participant‐specific 4D fMRI datasets that only contained changes in the intensity values within the bilateral hippocampus. We then performed group ICA on this spatially restricted dataset using FSL Melodic v3.15 with a specific number of dimensions. This specific number of dimensions was determined by an optimization procedure that maximized, across a range of different dimensions, the relationship between a set of obtained independent components (ICs) and the seven resting‐state networks found by Yeo et al., (2011). In other words, this procedure assumed that ICA produced a set of ICs where some of these components were associated with known resting‐state networks. The goal of the procedure was therefore to find the specific dimension that optimizes the relationship between a set of ICs and their corresponding resting‐state networks. The main advantage of this procedure is that it resolves the typical issues of deciding the dimensionality of the ICA and the subsequent determining of the status of obtained ICs as signal or noise in a single step (e.g. Salimi‐Khorshidi et al., 2014).

Specifically, this procedure involved the following three main operations. First, srICA was performed on the same dataset at dimensions ranging from 1 to 15 in a stepwise fashion. In the next step, whole‐brain group‐level FC maps corresponding to each IC in each dimension were obtained using Dual Regression (Nickerson et al., 2017). In the Dual Regression procedure, each IC map was first regressed against each participant's cleaned and denoised 4D fMRI data, and the resulting time courses for each IC map were then regressed for a second time against the same fMRI dataset. In the final step of Dual Regression, the regression coefficients of the whole‐brain participant‐specific FC maps were Fisher‐transformed into Z‐values. Whole‐brain group‐level FC maps for each dimension were then computed from the participant‐specific FC maps using a one‐sample group‐mean *t* test implemented in FSL randomise with default settings. These whole‐brain group‐level FC maps for each dimension were then correlated with the seven well‐known resting‐state networks found by Yeo et al., (2011) using the fslcc function from FSL. Given that the FC maps directly correspond to the different activation clusters inside the hippocampus detected by the srICA procedure (the ICs), the correlation between a given FC map and a given reference network therefore indicates the degree to which a given activation‐cluster participates in that reference network.

This produced 15 correlation‐matrices (one for each dimension) of size *n × m,* where *n* refers to the number of ICs and ranges from 1 to 15 and *m* refers to the number of reference resting‐state networks, here 7. In the final step, these 15 correlation matrices were each subjected to an algorithm that output an IC if its maximum correlation with a given resting‐state network was above a threshold (*r_max_* > 0.4) and if this maximum correlation was sufficiently higher than the second highest correlation ($\frac{r_{\max}}{r_{\max2}}$ > 1.3), both within the same IC and within the same resting‐state network. As an example, consider an srICA with a dimension of 2, and that the FC map (obtained with Dual Regression) corresponding to activation cluster denoted by IC0 correlates {0.1, 0.5, 0.2, 0.3, 0.2, 0.1, 0.1} with the seven resting networks and that the FC map corresponding to IC1 correlates {0.1, 0.1, 0.1, 0.2, 0.1, 0.3, 0.1} with these networks. In this case, the algorithm would output IC0 because its maximum correlation with resting‐state network 2 is higher than 0.4, and because this correlation is sufficiently different from the next highest correlation within the same IC (i.e. $\frac{0.5}{0.3}$ > 1.3) and within the same network (i.e. $\frac{0.5}{0.1}$ > 1.3). Note that parameters for the correlation threshold and the ratio were manually set at values that produced sensible results. In future studies, we will attempt to further automate this aspect of the procedure. Following the standard idea that ICA at larger dimensions produces more fractionated components, we then chose the lowest dimension at which this algorithm produced the largest number of ICs. This procedure therefore detected in a data‐driven fashion the optimal number of dimensions for which the srICA produced the largest number of clusters of voxels inside the hippocampus that showed both strong and unique correlations with known resting‐state networks.

#### 2.4.3 Group‐level analyses of whole‐brain FC

The goal in the next step of the analysis was to establish the statistical reliability of the co‐activity of the individual brain regions identified as connected to each activation‐cluster inside the hippocampus (i.e., the IC) across all participants. To this end, we first intersected the whole‐brain participant‐specific FC maps obtained with Dual Regression with the participant‐specific aparc + aseg atlas computed by FreeSurfer. We used 42 bilateral areas in the aparc + aseg atlas (excluding large areas like brainstem and cerebellum for which a single mean value may not be representative). We then extracted the mean Z‐score value for each area and for each participant. These data were then subjected to a regression model of the form:

(1) Z=hemisphere+FC_map×brain_region+rand(participant),

where *hemisphere* was a co‐factor with two levels (left versus right), *FC*_*map* was a discrete variable with number of levels equal to the number of ICs identified in the previous step, *brain*_*region* was a factor with number of levels equal to the sum of the number of cortical and subcortical regions in the aparc + aseg atlas (here 42) and *participant* was a random factor with number of levels equal to the total number of participants (i.e. 172). The dependent variable *Z* was the average Z‐value for the cortical and subcortical regions obtained from each participant's aparc+aseg file. Note that by modeling the participant as a random factor, we took into account individual participant variation in the estimation of the mean Z‐score value that provided us with more accurate estimates as opposed to simply averaging the mean Z‐score values across all participants. In addition, because the average Z‐scores were obtained from the areas in the participant‐specific Desikan–Killiany atlases, this is a volume‐based group‐analysis that takes into account the unique morphology of each participant's brain. It is therefore not subject to common concerns in volume‐based group analyses that assume that each participant's brain morphology is the same (see Anticevic et al., 2008; Glasser et al., 2013, for discussion of this issue).

The main interest in this model was in the interaction term *FC*_*map* × *brain*_*region*. This interaction provided a test of the null‐hypothesis that brain regions would be activated in the same way across the different FC maps. In the case that the interaction term was significant (defined as *p* < .05), we performed post‐hoc comparisons where we contrasted the co‐activity of each brain region with the mean of the co‐activity values of all other brain regions for each IC (i.e. an ‘effect’ contrast). This therefore produced a list of cortical and subcortical regions for each IC that showed reliable co‐activity with this IC relative to the mean of all other brain regions.

Mixed‐effect regression modeling relied on the lme4 package (v1.1.23; Bates et al., 2007) implemented in R (v4.0.0). ANOVA tables (Type III) were computed directly from the output of the mixed‐effect regression models using the lmerTest package (v3.1–2; Kuznetsova et al., 2017). *p*‐values in these models were calculated using the Satterthwaite correction for the degrees of freedom. Post‐hoc testing was performed using the emmeans package (v1.4.6; Lenth et al., 2018) when the interaction term was significant (*p* < .05). *p*‐values were adjusted for multiple comparisons using the Bonferroni method. Results were visualized using the ggseg (v1.5.4; Mowinckel & Vidal‐Piñeiro, 2019) and ggpubr packages (v0.3.0; Kassambara, 2018).

#### 2.4.4 Relative contributions of the hippocampal subfields

The goal in the final step of the analysis was to determine the relative contributions of the hippocampal subfields in the various putative resting‐state networks detected in previous steps. Specifically, we attempted to obtain a list that ranked each hippocampal subfield with respect to its relative contribution in the FC maps that represented the resting‐state networks. This was achieved by first intersecting the participant‐specific hippocampal subfield masks (described above) with each participant‐specific whole‐brain FC map. The resulting average Z‐values for each hippocampal subfield and each participant were then fitted to the same statistical model as described in Equation 1. In this model, the term *brain_region* now referred to the six hippocampal subfields. As before, our specific interest was in the interaction term of the model (*FC* *_map* *× brain_region*) that provided a test of the null‐hypothesis of whether the six hippocampal nuclei were activated in the same way across the various FC maps. However, the post hoc tests that were performed when this interaction term was significant differed from those described above. Specifically, in order to determine the relative contribution of the subfields to the different resting‐state networks, we first performed pairwise comparisons of all six hippocampal subfields within each FC map. This produced a list of 15 pairwise comparisons with a test statistic (i.e. the z‐ratio; see below) that reflected the degree to which the co‐activity of a given subfield differed from another subfield. These pairwise test‐statistics were then summed, ordered and thresholded at >0 for each hippocampal subfield to produce a ranked estimate of the relative contribution of each subfield to each putative resting‐state network connected to the hippocampus.

## 3 RESULTS

### 3.1 Detection of activation clusters inside hippocampus and relationship with resting‐state networks

The procedure for finding the optimal number of dimensions for the ICA returned that across Dimensions 1–15, Dimension 10 was the lowest dimension at which the largest number of ICs and their corresponding whole‐brain FC maps were strongly and uniquely connected to different resting‐state networks. Specifically, we found that for Dimension 10, two ICs were connected to different networks: IC0 was correlated *r* = 0.49 with the somatomotor network and IC1 was correlated *r* = 0.56 with the default mode network (see Table 1 for an overview of the correlations of these ICs with all networks, and see Table S1 for the results of a correlation analysis restricted to only those cortical voxels included in the reference networks, which yielded similar results). As can be seen in Supplementary Figure S1, strong correlations (*r* > 0.40) were frequently found for these two networks across all dimensions, suggesting that the detection of these two networks was not idiosyncratic to Dimension 10. In addition, as can be seen in Supplementary Figure S2, we also examined Dimensions 20 and 30, and this did not lead to the detection of new networks. We can therefore conclude that for our data, the specific clusters of voxels detected by the ICA using Dimension 10 for IC0 and IC1 were optimal in connecting with known resting‐state networks.

**TABLE 1:** Table of correlations of the FC maps with resting‐state networks

Correlation with FC maps		
Yeo et al., (2011), 7 networks	IC0	IC1
1 ‐ Visual	0.13	0.12
2 ‐ Somatomotor	0.49	0.27
3 ‐ Dorsal attention	0.05	0.02
4 ‐ Ventral attention and salience	0.00	0.03
5 ‐ Limbic	0.06	0.04
6 ‐ Executive control	0.02	0.17
7 ‐ Default mode	0.23	0.56

The grey shade indicates the highest correlation, both row and columnwise.

**FIGURE 2:**
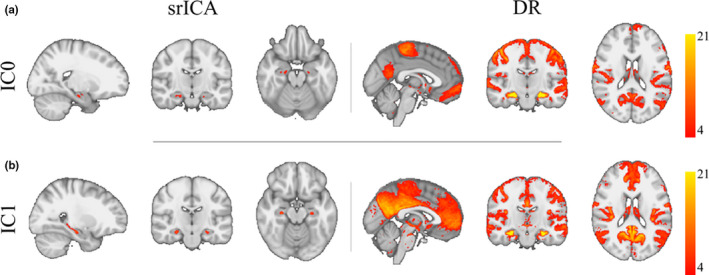
Results from group spatial ICA restricted to the hippocampal region (left panels under heading srICA) and Dual Regression (right panels under heading DR) in the two detected ICs (a,b). The maps under DR show whole‐brain group‐level FC maps using the hotspots detected using the ICs as seeds. Note that IC0 correlated with the somatomotor network and IC1 with the default mode network. IC and FC maps are corrected and thresholded Z maps at Z > 4

**FIGURE 3:**
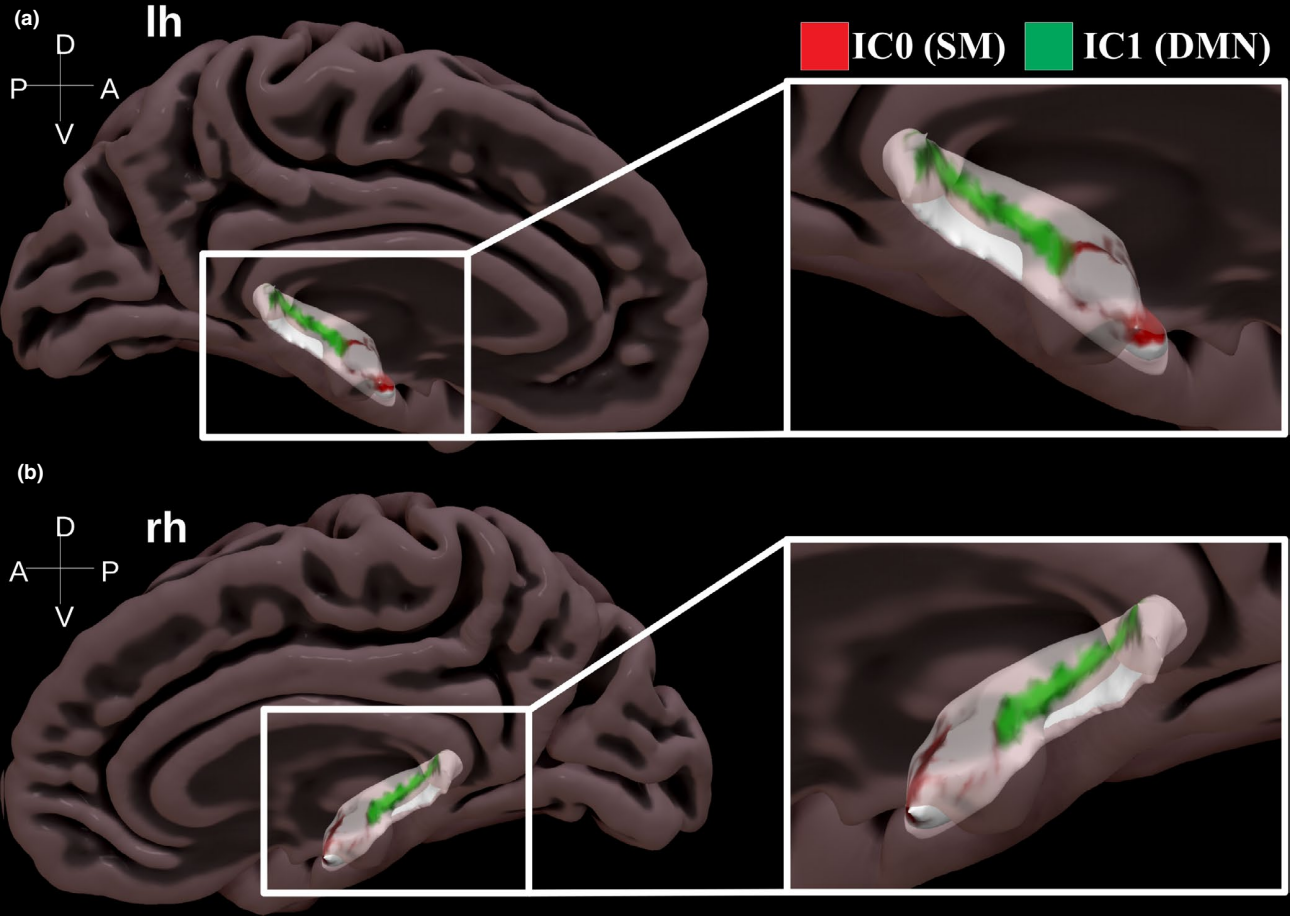
Surface projection of activity hotspots along the hippocampal long‐axis seen in a medial‐sagittal view for the left (a) and right hemisphere (b). Note how there is a bilateral activity hotspot in the anterior section of the hippocampus (IC0 correlated with somatomotor network, red colour) and a bilateral activity hotspot that extends from middle to posterior sections (IC1 correlated with default mode network, green colour)

A visual presentation of the location of these clusters of voxels along with their whole‐brain group‐level FC map computed with Dual Regression is presented in Figure 2. A full overview of all 10 ICs from Dimension 10 and their FC maps is shown in Supplementary Figure S3. In these whole‐brain FC maps (right column), it can be appreciated that the two ICs have relatively contrasting FC with the rest of the brain. In addition, a further visualization in surface space of the two ICs with their projected location on the hippocampal long‐axis is presented in Figure 3. Here, it can be seen that whereas IC0 (correlated with the somatomotor network), is located in a more anterior section of the hippocampus, IC1 (correlated with the default mode network) is located from middle to posterior locations along the hippocampal long‐axis.

### 3.2 Group‐level analyses of whole‐brain FC

**FIGURE 4:**
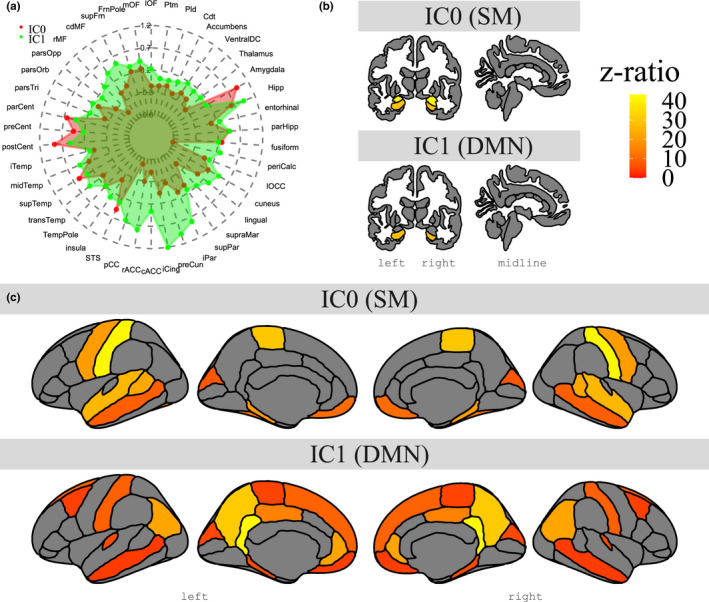
Overview of model‐derived estimated marginal means for whole‐brain group‐level FC for the two ICs in areas from the Desikan–Killiany atlas (a), as well as contrast effects in subcortical (b) and cortical structures (c), where IC0 shows the contrast of each region reliable more co‐activated than the mean co‐activity of all other regions within IC0 (top row), and IC1 shows the contrast of each region reliable more co‐activated than the mean co‐activity of all other regions within IC1 (bottom row). Note how IC0 connects to regions of the somatomotor network and how IC1 connects with regions of the default mode network

Group‐level analyses revealed those regions that were reliably co‐activated in the FC maps that corresponded to IC0 and IC1 (see Figure 4a for the [model‐derived] estimated marginal‐mean values for each region by IC). Specifically, Linear Mixed‐effect Regression analyses taking participant variability into account revealed a main effect of *H* *emisphere* (*F*(1,28,640) = 132.6, *p* <.0001), suggesting higher co‐activity values in the left versus the right hemisphere. In addition, there was a main effect of *B* *rain Region* (*F*(41,28,640) = 251.6, *p* <.0001), suggesting that co‐activity values differed between the different brain regions of the Desikan–Killiany atlas. Furthermore, there was a main effect of IC (*F*(1,28,640) = 4,659.4, *p* <.0001), suggesting that co‐activity values differed between the different FC maps. Important for our present purposes, there was a significant interaction between *B* *rain Region* and *IC* (*F*(41,28,640) = 209.7, *p* <.0001), suggesting that average co‐activity values for each brain region differed between the ICs.

Further exploration of this interaction with post‐hoc tests revealed the list of regions for a specific IC where one region was significantly more co‐activated compared with the mean co‐activity of all other regions. As can be seen in Table 2, IC0 and its corresponding FC map revealed regions typically associated with the somatomotor network like sensorimotor cortex (pre‐ and postcentral gyrus) and the amygdala. In addition, Table 3 showed that IC1 and its corresponding FC map had high co‐activity values in areas typically associated with the default mode network like posterior midline areas (isthmus cingulate, precuneus) and medial frontal areas (medial orbital frontal, frontal pole). A visual presentation of these results is shown in Figure 4b,c.

**TABLE 2:** Cortical and subcortical areas showing reliable co‐activity with IC0 (correlated with somatomotor network) relative to the mean co‐activity value of all other areas. *p*‐values corrected for multiple comparisons using Bonferroni correction

Region	Z‐ratio	*p*‐Value
Amygdala	41.94	<2.225E‐308
Postcentral	40.02	<2.225E‐308
Paracentral	29.07	3.672E‐184
Hippocampus	28.55	1.098E‐177
Superior temporal	25.49	9.978E‐142
Bankssts	22.64	8.040E‐112
Parahippocampal	20.08	4.427E‐88
Precentral	19.99	3.157E‐87
Fusiform	12.53	2.226E‐34
Medial orbitofrontal	10.72	3.553E‐25
Frontal pole	10.02	5.469E‐22
Temporal pole	8.82	5.069E‐17
Middle temporal	8.34	3.331E‐15
Cuneus	7.09	5.786E‐11

**TABLE 3:** Cortical and subcortical areas showing reliable co‐activity with IC1 (correlated with default mode network) relative to the mean co‐activity value of all other areas. *p*‐values corrected for multiple comparisons using Bonferroni correction

Region	Z‐ratio	*p*‐Value
Isthmus cingulate	40.55	<2.225E‐308
Precuneus	29.75	8.776E‐193
Hippocampus	27.56	1.373E‐165
Inferior parietal	21.71	6.866E‐103
Rostral anterior cingulate	21.54	2.931E‐101
Posterior cingulate	14.52	3.992E‐46
Superior frontal	8.85	3.841E‐17
Postcentral	8.57	4.487E‐16
Transverse temporal	5.79	3.020E‐07
Parahippocampal	5.72	4.762E‐07
Cuneus	5.12	1.351E‐05
Paracentral	4.39	4.955E‐04
Caudal middle frontal	4.20	1.181E‐03
Medial orbitofrontal	3.82	5.826E‐03
Middle temporal	3.63	1.267E‐02

### 3.3 Relative contributions of the hippocampal subfields

**FIGURE 5:**
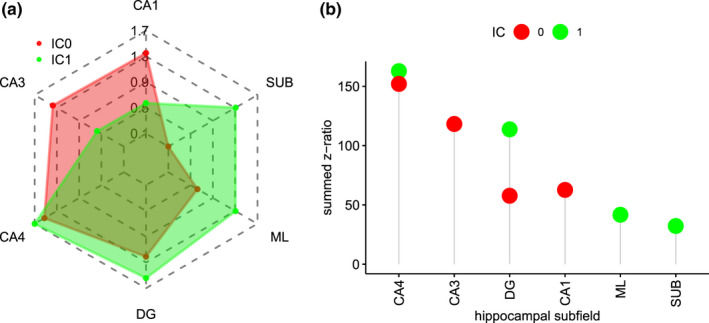
Group‐level co‐activity values for each hippocampal subfield in each IC from model‐derived estimated marginal means (a) and summed z‐ratios that indicate the strength of co‐activity of each subfield within a given resting‐state network (IC). Note how for IC0 (somatomotor, red colour), there is strong co‐activity for CA4/DG, CA1 and CA3, and how for IC1 (default mode, green colour), there is strong co‐activity in CA4/DG and SUB. Note also ML is listed for reasons detailed in the text

Statistical analyses of the relative contribution of the hippocampal subfields in the two FC maps revealed a main effect of *B* *rain Regio*n (*F*(5,3,944) = 1,183.8, *p* <.0001), suggesting that there were differences in co‐activity between the subfields. Furthermore, a main effect of *IC* was found (*F*(1,3,944) = 262.0, *p* <.0001), suggesting differences in co‐activity between the different ICs. Again, important for our present purposes, there was a significant interaction between *B* *rain Region* and *IC* (*F*(5,3,944) = 1,464.1, *p* <.0001), indicating that the subfields were not co‐activated in the same way across the different FC maps. Further exploration of this interaction using pairwise tests within each IC and then ranking the six subfields revealed the relative contribution of each hippocampal subfield. Specifically, as can be seen in Table 4, for IC0 (correlated with the somatomotor network) summed z‐ratios were in descending order ranked CA4, CA3, CA1 and DG. Similarly, Table 4 showed that for IC1 (correlated with the default mode network), summed z‐ratios were ranked CA4, DG and SUB (see also Figure 5a for the [model‐derived] estimated marginal mean values for each subfield by IC and Figure 5b for a visual presentation of the summed z‐ratios for each subfield by IC).

**TABLE 4:** Relative contributions of each hippocampal subfield within the different resting‐state networks (indicated by IC). Rankings based on the sum of pairwise z‐ratio differences for all substructures within a given IC

Subnucleus	IC	Summed z‐ratio	Rank
CA4	0	152.06	1
CA3	0	118.21	2
CA1	0	62.63	3
DG	0	57.68	4
CA4	1	162.81	1
DG	1	113.69	2
SUB	1	32.19	3

### 3.4 Complementary analyses

In two further analyses, we attempted to examine these results in more detail. First, we examined to what extent the co‐activity in the different subcortical and cortical areas found for the different ICs would depend on the hemisphere. To this end, we modelled the data using Equation 1 but also included the triple interaction term *hemisphere* *×* *FC_map* *×* *brain_region* to examine whether the *FC_map* *×* *brain_region* interaction would depend on the hemisphere. The results of this analysis revealed that the triple interaction between *hemisphere* *×* *FC_map* *×* *brain_region* was significant (*F*(41,28,557) = 4.92, *p* <.0001). As can be seen in Tables S3 and S4, generally, the same set of regions was detected between both hemispheres albeit with slight differences in detection accuracy (the full output of the regression is reported in Table S2). Given that the focus of our paper is not on hemispheric differences in hippocampal FC, we do not discuss this issue further.

In addition, we examined in further detail the areas that had different co‐activity between IC0 and IC1. Specifically, we performed post‐hoc tests on the same model from Equation 1 where for each area we now contrasted co‐activity from IC0 and IC1 (and vice versa) using pairwise post‐hoc tests that were corrected for multiple comparisons using the Bonferroni correction. As can be seen in Tables S5 and S6, the contrasts between these two ICs further highlighted the involvement of IC0 in the somatomotor network (predominantly activity in primary sensorimotor areas) and IC1 in the default mode network (strong activity along midline regions).

## 4 DISCUSSION

In the current study, we attempted to discover the precise anatomical location of hippocampal activity hotspots during the resting state, the resting‐state networks that are functionally connected to these hotspots and the configurations of hippocampal subfield activities that underlie these hotspots. To do this, we relied on data‐driven analysis techniques applied to the high spatial‐resolution 7T resting‐state fMRI dataset from the HCP. We found that group spatial ICA restricted to the hippocampus revealed two activity hotspots at different points along the hippocampal long‐axis. These two activity hotspots were functionally connected with two different sets of brain regions. One activity hotspot was located in an anterior section along the hippocampal long‐axis and connected with anterior areas like the amygdala and orbitofrontal cortex, as well as with sensorimotor cortex and medial and lateral temporal areas. This network was highly correlated with the well‐known somatomotor network (*r* = 0.49). Another hotspot consisted of an extended area of activities that spanned from middle to posterior locations along the hippocampal long‐axis and connected mainly with posterior midline areas like the posterior cingulate, precuneus and retrosplenial cortex (isthmus cingulate), as well as with medial orbitofrontal and inferior parietal cortex. This network was found to correlate with the well‐known default mode network (*r* = 0.56). Furthermore, we found that these two activity hotspots associated with different resting‐state networks were composed out of different hippocampal subfield configurations. Specifically, the somatomotor network relied strongly on activity in the CA4, DG, CA1 and CA3 subfields (and less on SUB), whereas the default mode network relied mostly on activity in the CA4, DG and SUB subfields (and less on CA1 and CA3).

These results clarify the exact section of the hippocampus that plays a role in the default mode network. Although previous studies had demonstrated that the hippocampus is a pertinent node in the default mode network, studies had reported inconsistent evidence regarding the precise section of the hippocampus that fulfills this role (Blessing et al., 2016; Kahn et al., 2008; Qin et al., 2016; Vincent et al., 2006). The current results suggest that a rather large section of the hippocampus that spans from middle to posterior locations along the hippocampal long‐axis is co‐activated with regions of the default mode network. These results are in line with anatomical connectivity studies in rodents that have found that default mode regions like the retrosplenial cortex project mostly to posterior regions in the hippocampus (Rosene & Van Hoesen, 1977; Strange et al., 2014) and with FC studies in humans that have found that posterior seeds in the hippocampus connected with default mode regions like the posterior cingulate and the precuneus (Adnan et al., 2016; Barnett et al., 2019; Kahn et al., 2008; Qin et al., 2016; Voets et al., 2014). However, our results seem at odds with previous studies that have concluded that seeds in anterior sections of the hippocampus connected to default mode regions (Blessing et al., 2016; Chase et al., 2015; Robinson et al., 2015; Vincent et al., 2006). Although the current study was not designed to resolve this discrepancy, we speculate that a combination of issues related to low spatial resolution and spatial smoothing to move signal from hippocampal body to anterior sections as well as uncertainties in classifying medial frontal regions as part of the default mode network versus the somatomotor network could have led to these conclusions. Clearly, further targeted studies need to be conducted to resolve this issue. In sum, the current results provide further information about the precise anatomical location of hippocampal activities that connect to the default mode network and further underscore the idea that the hippocampus is functionally organized along its long‐axis (Fanselow & Dong, 2010; Poppenk et al., 2013; Strange et al., 2014).

The current data also indicated that besides the default mode network, the hippocampus was connected to a second resting‐state network. Specifically, the anterior hotspot revealed a whole‐brain FC map that was strongly and uniquely correlated with the somatomotor network (*r* = 0.49; Yeo et al., 2011). The somatomotor network is a mostly cortical network that includes primary and sensory‐motor areas as well supplementary motor areas (Yeo et al., 2011). The observation of the hippocampus as connected with this somatomotor network makes sense in the context of what is currently known about hippocampal function. Specifically, current research points to a role for the hippocampus, and especially its CA1 and CA3 subfields, in the processing of discriminatory sensory information (Bakker et al., 2008; Hainmueller & Bartos, 2020; Pereira et al., 2007; Yassa et al., 2011). Why the sensory‐motor areas preferentially connected to the anterior section of the hippocampus is less clear. Connectivity of the anterior hippocampus is typically associated with an anterior hippocampal network that includes amygdala, orbitofrontal cortex and regions in the anterior temporal lobe (Aggleton, 2012; Ranganath & Ritchey, 2012). This network has been associated with the processing of perceptual, semantic and affective properties of items (Ritchey et al., 2015). In line with this proposal, our results also showed FC between the anterior hippocampus and the amygdala, orbitofrontal areas and temporal pole (see Table 2). The current results therefore suggest that in addition to the aforementioned regions, this anterior hippocampal network also has strong FC with primary sensory areas. Overall, the current results highlight the role of the hippocampus in the processing of sensory‐motor information and that moreover, the hippocampus plays a role in the somatomotor as well as the default mode network.

Furthermore, the current results revealed that these two different resting‐state networks relied on different contributions from the individual hippocampal subfields. Specifically, the results showed that the anterior hotspot that was connected to the somatomotor network depended primarily on activity in the CA1, CA3 and CA4/DG subfields (and less on SUB) and that the middle/posterior hotspot that was part of the default mode network depended primarily on SUB and CA4/DG subfields (and less on CA1 and CA3). These results are consistent with anatomical studies that have shown that the CA1 subfield connected more to anterior areas like the amygdala, orbitofrontal cortex and nucleus accumbens, as well as medial and lateral temporal areas, and that the SUB subfield was more connected to posterior midline areas such as the retrosplenial cortex, anterior thalamic nuclei and mammillary bodies (Aggleton, 2012; Rosene & Van Hoesen, 1977). Similarly, these results are in line with previous FC studies that have shown that posterior pre‐ and para‐subiculum were connected to retrosplenial cortex (Dalton et al., 2019) and that CA1 was connected with the amygdala (de Flores et al., 2017). Taken together, the current results suggest that different resting‐state networks depend on different configurations of hippocampal subfield co‐activity and that there are dissociations in connectivity between the CA1 and CA3 subfields on the one hand and SUB on the other (Aggleton, 2012).

The observation that different resting‐state networks depended on different configurations of hippocampal co‐activity raises the question of why this may be the case. One possible explanation is in terms of a difference in the degree to which these two networks rely on the processing of externally generated sensory information. Specifically, the somatomotor network involves activity in the primary sensory‐motor cortex and consequently suggests that this network is involved in the processing of externally generated sensory information. By contrast, the default mode network is typically considered to reflect processing of endogenous information (Buckner & DiNicola, 2019). Thus, in line with existing ideas about the role of the CA subfields in terms of processing incoming sensory information described above (Bakker et al., 2008; Hainmueller & Bartos, 2020; Pereira et al., 2007; Yassa et al., 2011), our results suggest that the CA subfields may be more functionally relevant for the processing of external incoming sensory information, whereas the SUB subfield is especially relevant for the processing of internally generated information. Future task‐based studies that place different demands on internal versus external generated information may shed further light on this issue.

Before concluding, one final issue deserves attention. A recent set of studies has examined the FC between the hippocampus and the rest of the brain using a new technique called Connectopic Mapping (Haak et al., 2018; Margulies et al., 2016; Przeździk et al., 2019; de Wael et al., 2018). This technique addresses a fundamental limitation of the ICA technique that assumes that the observed data reflect a mixing of underlying sources in a linear manner (Friston, 1998). Instead, the new technique does not make such an assumption and is able to detect non‐linear shapes (manifolds) in a similarity matrix representation of the data (Haak et al., 2018). Consistent with other studies mentioned earlier (Strange et al., 2014), these studies have observed that there are differences in the connectivity between the anterior and posterior sections of the hippocampus. However, these studies also show that this connectivity does not change abruptly along the hippocampal long‐axis but instead changes gradually in a smooth manner. These results are consistent with the results observed here. Specifically, our results show that within this smooth gradient of connectivity along the hippocampal long‐axis, there are particular hotspots that become activated during the resting state and that connect to specific brain networks. A combination of spatially‐restricted ICA used here and Connectopic Mapping could be used to provide further insight into the precise connectivity between activity hotspots in the hippocampus and the rest of the brain.

To conclude, the current study used a data‐driven technique with high spatial‐resolution 7T resting‐state fMRI data with 172 participants to explore the FC of the hippocampus during the resting state. We found that there were two activity hotspots in the hippocampus: One that occupied an anterior section along the hippocampal long‐axis, and second one that extended from middle to posterior locations along the hippocampal long‐axis. These two activity hotspots inside the hippocampus were connected to two known resting‐state networks: The somatomotor network and the default mode network. Finally, our results revealed that these two resting‐state networks relied on different hippocampal subfield configurations: Whereas the somatomotor network relied strongly on CA1, CA3 and CA4/DG fields and less on SUB, the default mode network relied strongly on SUB and CA4/DG fields and less on CA1 and CA3. These results therefore clarify the exact section of the hippocampus that connects to the default mode network, show that the hippocampus is connected to two distinct resting‐state networks, and that these two different resting‐state networks rely on different configurations of hippocampal subfield co‐activity. One avenue for future research is to determine whether these specific configurations of hippocampal subfield activity are affected by particular task‐based settings and whether they can serve as a biomarker for pathology in clinical settings.

## CONFLICT OF INTEREST

The authors have no conflicts of interest to declare.

## AUTHOR CONTRIBUTIONS

LE, NJ, EP and SS designed the study. LE, NJ and JAH analysed the data. LE and NJ wrote the manuscript.

### PEER REVIEW

The peer review history for this article is available at https://publons.com/publon/10.1111/ejn.15213.

## Supporting information

Supplementary Material

## Data Availability

The data that support the findings of this study are openly available from Human Connectome Project (www.humanconnectome.org).
