# Supplementary material for: Functional connectivity of the hippocampus and its subfields in resting‐state networks
Source: Eur J Neurosci. 2021 May 5;53(10):3378–93. doi: 10.1111/ejn.15213 (PMC8252772; doi:10.1111/ejn.15213)
Supplement: Supplementary file 1 — Supplementary Material [file EJN-53-3378-s001.pdf]

Supplementary Materials for Functional connectivity of the hippocampus and its subfields in resting-state networks

Laura Ezama[1,3,4], Juan A. Hernández-Cabrera[1,4,5], Sara Seoane[1,3,4], Ernesto Pereda[2,3,4] and Niels Janssen[1,3,4]

[1]Facultad de Psicología, Universidad de la Laguna, Spain

[2]Facultad de Ingeniería Industrial, Universidad de La Laguna, Spain

[3]Instituto de Tecnologías Biomédicas, Universidad de La Laguna, Spain

[4]Instituto Universitario de Neurociencia, Universidad de la Laguna,Spain

[5]Basque Center on Cognition Brain and Language, San Sebastián, Spain

Corresponding Author:
Niels Janssen
Tel: +34 922317502
e-mail: njanssen@ull.es

Tables

Table S1: Table of correlations of the FC maps with resting-state networks restricted to cortical voxels. Note these correlations are slightly higher.

Correlation with FC maps		
Yeo et al. (2011), 7 networks	IC0	IC1
1 - Visual	0.09	0.08
2 - Somatomotor	0.53	0.24
3 - Dorsal attention	0.04	0.01
4 - Ventral attention and salience	0.01	0.00
5 - Limbic	0.03	0.02
6 - Executive control	0.02	0.14
7 - Default mode	0.22	0.60

Table S2: ANOVA table displaying the results of the analysis that incorporates hemisphere into the regression model.

	Sum Sq	Mean Sq	NumDF	DenDF	F value	Pr(>F)
Hemisphere	20.28	20.28	1	28557	123.19	0.0000
Brain Region	1744.46	42.55	41	28557	258.41	0.0000
FC	758.00	758.00	1	28557	4603.68	0.0000
Hemi:Brain Region	43.55	1.06	41	28557	6.45	0.0000
Hemi:FC	2.72	2.72	1	28557	16.54	0.0000
Brain Region:FC	1439.56	35.11	41	28557	213.25	0.0000
Hemi:Brain Region:FC	33.21	0.81	41	28557	4.92	0.0000

Table S3: Cortical and subcortical areas showing reliable co-activity with IC0 for the left and right hemisphere.

Hemisphere	Region	Z-ratio	p-value
Left	amygdala	27.01	4.233E-159
	postcentral	25.08	3.325E-137
	paracentral	19.00	7.429E-79
	bankssts	18.09	1.520E-71
	hippocampus	17.18	1.571E-64
	superior temporal	17.16	2.247E-64
	precentral	12.48	4.194E-34
	parahippocampal	11.17	2.398E-27
	medial orbitofrontal	7.58	1.484E-12
	frontal pole	7.55	1.865E-12
	middle temporal	7.07	6.669E-11
	fusiform	6.56	2.320E-09
	inferior parietal	5.54	1.284E-06
	pars orbitalis	4.98	2.689E-05
	temporal pole	4.84	5.437E-05
Right	amygdala	31.35	4.493E-214
	postcentral	30.58	9.371E-204
	hippocampus	22.33	8.339E-109
	paracentral	21.24	1.791E-98
	superior temporal	18.04	4.055E-71
	parahippocampal	16.41	7.067E-59
	precentral	14.96	5.356E-49
	bankssts	13.08	1.758E-37
	fusiform	10.38	1.260E-23
	temporal pole	6.87	2.711E-10
	medial orbitofrontal	6.81	4.041E-10
	cuneus	6.56	2.263E-09
	frontal pole	5.85	2.016E-07
	middle temporal	3.97	3.083E-03

Table S4: Cortical and subcortical areas showing reliable co-activity with IC1 for the left and right hemisphere.

Hemisphere	Region	Z-ratio	p-value
Left	isthmus cingulate	29.20	7.206E-186
	precuneus	22.06	3.090E-106
	hippocampus	19.72	5.709E-85
	inferior parietal	17.01	2.727E-63
	rostral anterior cingulate	15.50	1.514E-52
	posterior cingulate	9.91	1.579E-21
	superior frontal	6.02	7.435E-08
	postcentral	5.06	1.717E-05
	parahippocampal	3.77	6.842E-03
	caudal middle frontal	3.31	3.858E-02
Right	isthmus cingulate	27.20	2.637E-161
	precuneus	19.13	6.204E-80
	hippocampus	18.39	6.390E-74
	rostral anterior cingulate	14.13	9.908E-44
	inferior parietal	12.86	3.120E-36
	posterior cingulate	9.83	3.397E-21
	postcentral	6.30	1.248E-08
	superior frontal	5.74	3.978E-07
	parahippocampal	3.57	1.477E-02
	cuneus	3.47	2.219E-02
	medial orbitofrontal	3.45	2.384E-02

Table S5: Areas with higher co-activity in IC0 compared to IC1.

Region	Z-ratio	p-value
amygdala	25.70	1.065E-145
postcentral	11.39	4.532E-30
precentral	7.16	8.133E-13
paracentral	6.69	2.235E-11
superior temporal	6.41	1.424E-10
bankssts	5.85	4.964E-09
fusiform	2.51	1.207E-02

Table S6: Areas with higher co-activity in IC1 compared to IC0.

Region	Z-ratio	p-value
posterior cingulate	42.93	< 2.225E-308
isthmus cingulate	40.18	< 2.225E-308
precuneus	39.07	< 2.225E-308
rostral anterior cingulate	35.62	6.047E-278
pericalcarine	35.44	4.690E-275
caudal anterior cingulate	28.68	7.235E-181
rostral middle frontal	25.77	1.978E-146
inferior parietal	23.80	3.148E-125
supramarginal	21.77	4.733E-105
superior frontal	18.21	4.089E-74
caudal middle frontal	17.39	1.040E-67
insula	15.22	2.731E-52
pars opercularis	12.81	1.422E-37
transverse temporal	12.48	9.050E-36
lateral orbitofrontal	12.48	1.009E-35
pars triangularis	11.49	1.502E-30
lingual	10.37	3.368E-25
hippocampus	9.76	1.608E-22
caudate	9.53	1.535E-21
cuneus	9.08	1.069E-19
ventral DC	7.95	1.884E-15
middle temporal	7.18	6.982E-13
entorhinal	6.31	2.712E-10
medial orbitofrontal	5.66	1.512E-08
putamen	5.44	5.281E-08
pars orbitalis	4.73	2.250E-06
pallidum	4.15	3.388E-05
frontal pole	3.87	1.099E-04
lateral occipital	3.84	1.226E-04
superior parietal	3.78	1.551E-04
inferior temporal	3.12	1.799E-03

Figures

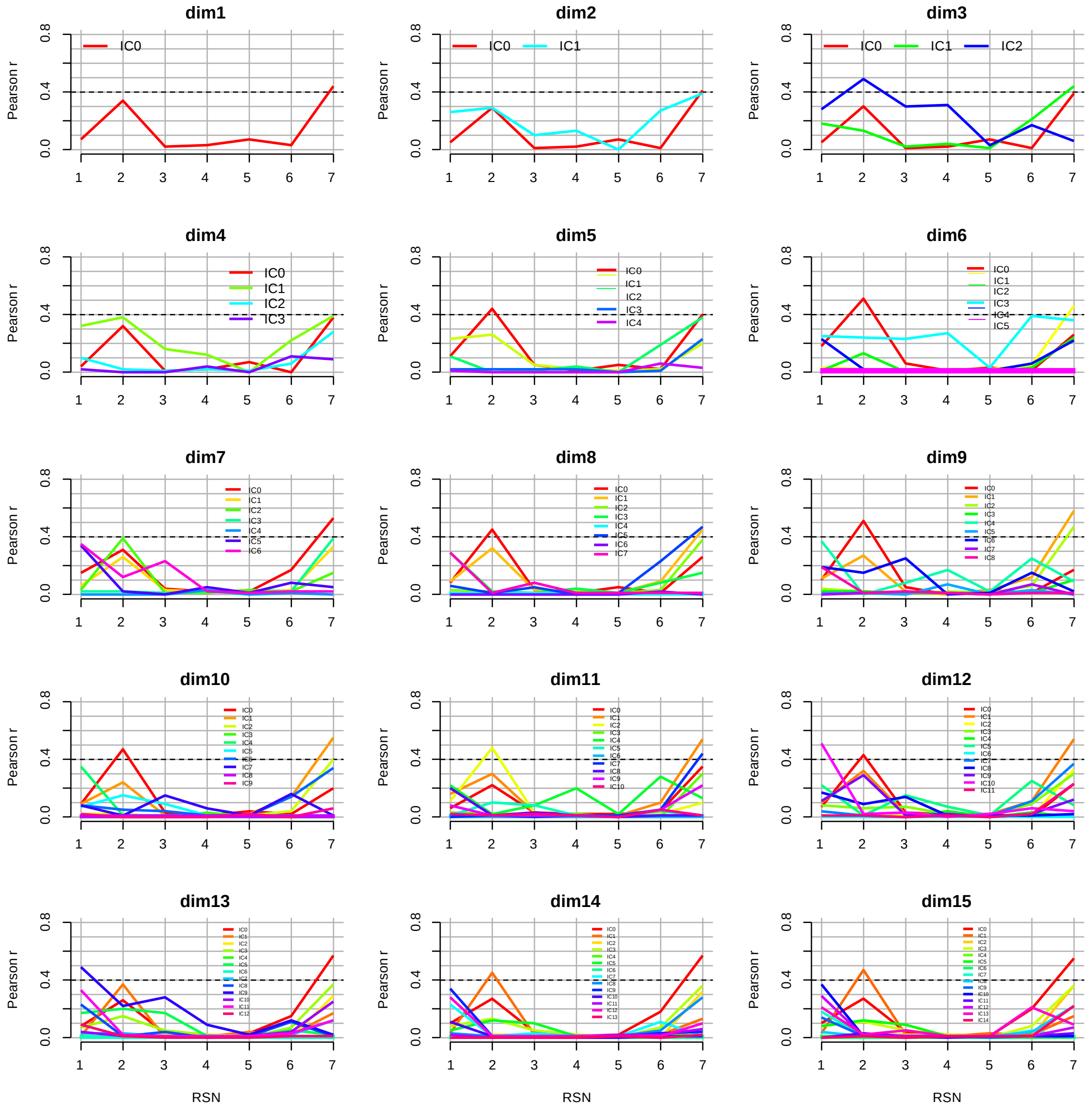

Figure S1: Correlations between independent components derived from srICA at dimensions 1 to 15 and the 7 different resting-state networks. Note how for most dimensions, ICs (and their corresponding whole-brain group level FC maps) show strong correlation (r > 0.4) with either detect network 2 (somatomotor) or 7 (default mode), and that dimension 10 is the lowest dimension where both ICs show strong and unique correlations (one IC correlates sufficiently with one and only one network, one network correlates sufficiently with one and only one IC) with the resting-state networks. See main text for further details.

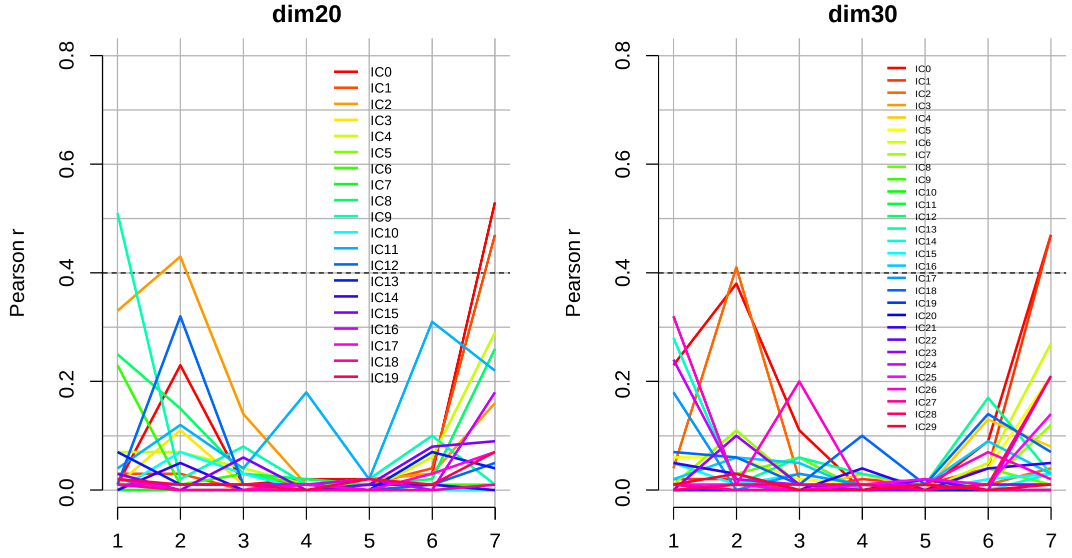

Figure S2: Correlations between independent components derived from srICA at dimensions 20 and 30 with the 7 different resting-state networks. Note how also for these higher dimensions, ICs (and their corresponding whole-brain group level FC maps) show strong correlation (r > 0.4) with either detect network 2 (somatomotor) or 7 (default mode). See main text for further details.

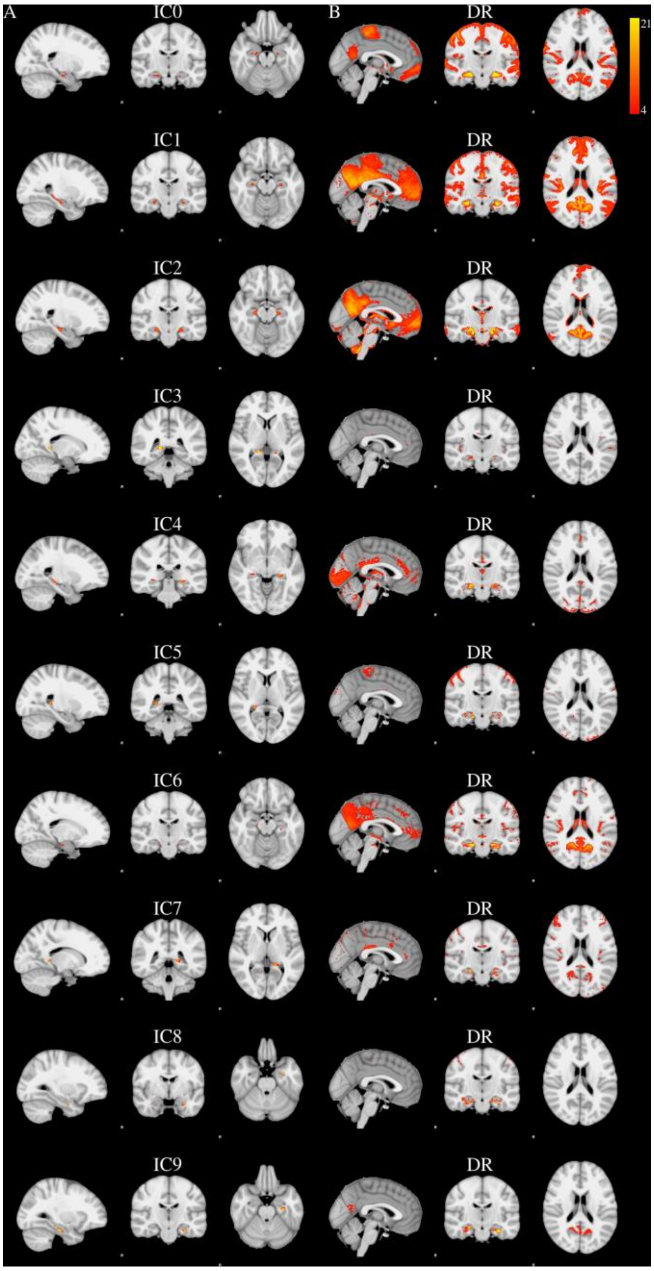

Figure S3: Overview of results from spatially restricted group ICA (left column) and Dual regression (right column) using dimension = 10 for all detected ICs in the HCP dataset. IC0 and IC1 were identified as having strong and unique correlations with resting-state networks. Red colors refer to low t-values and yellow colors to high t-values. Note how IC2 looks like default mode network but has activation in non-gray matter regions like ventricles. Note also how such artefacts can only be detected when no gray matter masks are used.
